# Who mimics nature? Disciplinary asymmetry in biomimetic and bioinspired research teams

**DOI:** 10.1098/rsif.2026.0324

**Published:** 2026-10-07

**Authors:** Jindong Zhang, Simon Baeckens, Raoul Van Damme, Kirsten Wommer, Kristina Wanieck

**Affiliations:** ^1^ Working Group Biomimetics and Innovation, Faculty of Applied Informatics, Campus Freyung, Deggendorf Institute of Technology, Freyung, Bayern, Germany; ^2^ Department of Biology, Laboratory of Functional Morphology, University of Antwerp, Antwerp, Flanders, Belgium; ^3^ Earth and Life Institute, UCLouvain, Louvain-la-Neuve, Belgium

**Keywords:** biomimicry, bio-inspired design, author affiliation classification, biologists, engineers, interdisciplinary, team composition

## Abstract

Biomimetics is generally viewed as inherently interdisciplinary, yet the disciplinary composition of biomimetic research teams has not been systematically quantified. We analysed 74 407 biomimetics-related publications from the past two decades and classified 436 010 author–paper affiliation records by biological or technical expertise to reconstruct team composition. Our analysis reveals an asymmetric disciplinary landscape: technical authors outnumber biological researchers by roughly two to one. Team organization is more balanced—while purely technical teams still represent the largest share of publications, interdisciplinary Bio–Tech teams have expanded over 20-fold since 2004 and now nearly match purely technical teams in annual output. Pathways to interdisciplinarity vary geographically: countries achieve Bio–Tech collaboration through distinct organizational models, from domestic multi-institutional networks to international partnerships. Interdisciplinary teams also achieve higher citation performance than teams composed exclusively of biological or technical researchers. Team composition is further associated with biological models used for inspiration: purely biological teams draw on a broader range of taxa, whereas studies involving technical researchers rely more often on a narrower set of conventional organisms and typically report their biological models with lower taxonomic specificity. These findings map the team architecture of biomimetics and support more targeted approaches to interdisciplinary collaboration.

## 1. Introduction

Biomimetics seeks to translate biological knowledge into technical innovations for solving practical problems [1,2]. In this manuscript, we use ‘biomimetic research’ in an inclusive bibliometric sense to refer to the combined literature on biomimetics, biomimicry and bioinspired research. Although biomimetics, in a stricter sense, involves the abstraction and transfer of biological principles into technical applications [1], bioinspiration is often used more broadly for research and design guided by biological systems [3,4]; in publication practice, however, these labels overlap substantially and are applied inconsistently [5]. Whether framed as biomimetics or bioinspiration, their processes necessarily cross disciplinary boundaries: they connect explanations of form, function and evolution in living systems to design, engineering and physical implementation [2–4]. The field is therefore routinely described as ‘inherently interdisciplinary’, and numerous studies describe cross-disciplinary collaboration as both a defining feature and a prerequisite for biomimetic success [4,6,7]. Yet, one key question remains: to what extent is biomimetic research interdisciplinary?

The question is not rhetorical. Interdisciplinarity in biomimetics is widely advocated, but rarely quantified. We know little about how often biological and technical expertise co-occur within the same research teams, or how large the pool of biologically trained contributors actually is relative to physical and engineering scientists. What evidence does exist paints a partial picture: using a three-month Web of Science snapshot of approximately 300 publications, Snell-Rood [8] reported in 2016 that fewer than 8% of biomimetic papers listed an author based in a biology department, an indicator of how rarely biologists appear as visible contributors. Building on this concern, Graeff *et al.* examined how biologists’ input shapes biomimetic design outcomes and called for workflows that explicitly integrate biological expertise in design teams [9–11]. Complementary evidence from the commercial side is consistent: in the BioM Innovation Database of commercial and pre-commercial biomimetic products, teams with at least some disciplinary diversity were more likely to meet true biomimetic criteria than mono-disciplinary ones [12]. Combined, these studies converge on a common message: biological expertise matters for the quality of biomimetic outcomes, including broadening the selection of usable biological models and improving the interpretation of biological function [9,11,13]. Yet, an in-depth analysis of the disciplinary composition of biomimetic teams and their evolution over time is still lacking. One dimension that makes this gap especially consequential is the choice of biological models. Recent evidence shows that biomimetic research draws on a relatively narrow subset of organisms, leaving much of biodiversity's functional repertoire unexplored [14]. Whether this taxonomic concentration is related to the disciplinary background of the researchers involved has not been examined.

Beyond the interdisciplinarity narrative, several researchers have argued for a more transdisciplinary framing of biomimetics, in which collaboration extends not only across academic disciplines, but also to industrial and societal actors who co-shape design briefs and implementation contexts [15,16]. In that perspective, the challenge is not simply to ‘add a biologist’ to an engineering project, but to organize sustained knowledge exchange between biological and technical domains and translate it into application-oriented teams. Related work on biologically inspired innovation has further emphasized that this translation is often constrained by the gap between academic research and industrial implementation, including the broader ‘valley of death’ between concept development and commercialization [17]. Conceptual and empirical studies have begun to examine such role distributions and knowledge-transfer processes in individual biomimetic projects [8,10,11]; however, these studies are based on reasonably small sets of case studies.

Here, we provide the first large-scale quantitative overview of the disciplinary composition of biomimetic research teams. Using a dataset of 74 407 biomimetics-related publications obtained from the Web of Science spanning two decades (2004–2025), we analyse the disciplinary background of a total of 436 010 author–paper affiliation records and reconstruct the composition of biomimetics research teams. We then link team composition to several key axes of biomimetics research, from its temporal expansion and collaboration geography to topical distribution, citation metrics and breadth of biological model use. This allows us to address three questions: first, which disciplines contribute to biomimetics research, and how has their relative presence changed over the past two decades? Second, is interdisciplinary team composition associated with differences in citation performance and topical specialization? Third, is the presence of biological expertise associated with differences in the diversity and taxonomic specificity of the organisms used as sources of inspiration in biomimetic research?

## 2. Material and methods

### 2.1. Dataset construction and affiliation extraction

We queried the Web of Science (WoS) Core Collection on 13 November 2025 using the topic search string ‘TS = (biomim* OR bio*inspir*)’ with no restriction on document type, yielding 74 407 records published between 2004 and 2025. This search strategy was chosen to capture the broader publication landscape of biomimetic, biomimicry and bioinspired research as completely as possible. The corpus therefore comprised publications whose title, abstract, keywords or other WoS topic-indexed fields contained biomim* terms (e.g. biomimetic, biomimetics, biomimicry) or bio*inspir* terms (e.g. bioinspired, bio-inspired, bioinspiration). We did not, however, verify whether each article fell strictly within the scope of biomimetics; this should therefore be considered when interpreting the results. WoS encoded author-specific affiliations in the ‘Addresses’ field through bracketed blocks (e.g. [Author 1; Author 2] affiliation text; [Author 3] affiliation text). A custom Python pipeline (supplementary material, S1) parsed these blocks and returned, for each paper, a set of author–paper affiliation records. Because each of such records linked one author to one paper, the same individual contributing to multiple publications appeared as multiple records. When no bracket structure was present, affiliations could not be linked to individual authors; therefore, the full ‘Addresses’ and ‘Reprint Addresses’ field was retained for paper-level analyses only.

Affiliation strings contained heterogeneous components (e.g. names of research units, departments, institutes, street addresses, postal codes). To maximize signal for disciplinary inference while reducing noise, each affiliation block was segmented, filtered to remove non-informative tokens and normalized through deterministic substitution rules (abbreviation expansion, multilingual harmonization). The full segmentation, filtering and normalization logic is documented in supplementary material, S1.

We used the WoS Core Collection because it provided structured, exportable metadata required for the present team-composition analysis, including author-address and affiliation fields, subject categories, research areas, DOI information and citation fields. Other databases, including Google Scholar, might provide broader coverage, but did not offer comparable bulk export or consistently structured author-affiliation metadata for this type of analysis. The present results should therefore be interpreted as applying to WoS-indexed scholarly publications; using a different database would be expected to affect the retrieved corpus and might consequently influence some quantitative estimates.

### 2.2. Disciplinary classification of affiliations

Normalized affiliation segments were assigned to one or more of six disciplinary categories (table 1). Four of the key categories were aggregated into broad groups for further analysis: Life-Sciences (BIO and MED) and Technical-Sciences (ENG and PHYS–CHEM). This aggregation was motivated by the central aim of the study—to examine how biological and technical expertise were represented and combined within biomimetics research teams. In this context, MED was grouped with BIO because it reflected life-science expertise grounded in the study of living systems, whereas PHYS–CHEM was grouped with ENG because these fields typically contributed to the design, fabrication or physical characterization of technical solutions.

**Table 1.** Disciplinary categories used to classify author affiliation segments. Each normalized segment was matched against a hierarchical set of keyword patterns; a segment could trigger more than one category.

broad group	disciplinary category	description
life-sciences	BIO	biological sciences, including ecology, zoology, botany, genetics, molecular and cell biology, microbiology, biochemistry, biophysics, agriculture, forestry, fisheries and food science
	MED	health sciences, including medicine, dentistry, veterinary medicine, pharmacy, public health and clinical medicine
technical-sciences	ENG	engineering, computer science, materials science and closely related technological fields
	PHYS–CHEM	physics and non-biological chemistry, including applied physics, physical chemistry, analytical chemistry and related fields, when not already captured under ENG
–	OTHER	architecture, design, arts, humanities, social sciences, economics, mathematics, applied mathematics, statistics and environmental or earth sciences
–	UNKNOWN	segments that did not contain sufficient information to identify the field of research, such as affiliations listing only a university name without a department or sub-unit

An author was assigned to every category for which the affiliation contained matching keywords, so that a single author could carry multiple labels (e.g. an author affiliated with both a biology and an engineering department would be labelled BIO + ENG). Hybrid field labels were treated in the same way: affiliation terms such as ‘biological engineering’, ‘bioengineering’ or ‘biomedical engineering’, and common abbreviations such as ‘biomed engn’ were assigned to both BIO and ENG, because they explicitly combined biological and engineering domains. These cases therefore contributed to the hybrid Life-Sciences and Technical-Sciences author profiles used in the team-type classification. The classification followed a multi-layer hierarchical rule-based pipeline implemented in Python to ensure that (i) explicit, auditable rules controlled known edge cases, while (ii) generalizable patterns covered the majority of affiliation strings. The classification method, including the complete rule sets, keyword lists and processing logic, is provided in supplementary material, S1.

### 2.3. Team-type classification and publication trends

In this study, we treated all authors listed for a given paper as a single research team; accordingly, team types were defined at the paper level. Four mutually exclusive paper-level team types were defined (table 2). Here, ‘Bio’ was used as a shorthand for the aggregated Life-Sciences group (BIO + MED), whereas ‘Tech’ denoted the aggregated Technical-Sciences group (ENG + PHYS–CHEM).

**Table 2.** Paper-level team types.

team type	definition
Bio–Tech	≥1 Life-Sciences author AND ≥1 Technical-Sciences author
Tech-only	≥1 Technical-Sciences author AND no Life-Sciences author
Bio-only	≥1 Life-Sciences author AND no Technical-Sciences author
Other/Unknown	all authors carry only OTHER/UNKNOWN labels

The Other/Unknown team type was assigned only when no author on the paper carried a Life-Sciences or Technical-Sciences label. Thus, whenever at least one author had a Life-Sciences or Technical-Sciences label, the paper was classified on that basis, regardless of how many co-authors remained in OTHER or UNKNOWN. A fallback mechanism was applied to papers for which no author-affiliation-based classification was possible: discipline was inferred from the WoS ‘Categories’ and ‘Research Areas’ fields. Classifications derived from this fallback were flagged with a question-mark suffix (e.g. ‘Tech-only?’) to distinguish them from author-affiliation-based assignments; they were reported descriptively but excluded from the primary statistical analyses. For descriptive summaries of field-wide team composition, these uncertain fallback classifications were grouped with Other/Unknown teams, so that they could be retained for completeness without being interpreted as confidently assigned Bio–Tech, Bio-only or Tech-only teams.

To test whether publication growth differed among team types, we fitted a negative binomial regression on annual publication counts with team category, centred publication year (Year – 2004; centred at the first year in the corpus) and their interaction as independent factors. A negative binomial specification was chosen because a likelihood-based overdispersion test rejected the equidispersion assumption of the Poisson model (*α* = 0.003, *p* < 0.001). Team was coded with Bio–Tech as the reference category. The Year × TeamType interaction tested whether annual growth rates differed among team types; significance was assessed by a likelihood-ratio test, comparing the full model with a reduced model without the interaction. Exponentiated coefficients were reported as incidence rate ratios (IRRs).

### 2.4. Author roles and collaboration structure

To examine who occupied key positions in Bio–Tech teams, we used the order of names in the WoS ‘Author Full Names’ field to identify first and last authors, as authors in these positions were typically main contributors to a research paper in the fields considered [18,19]. Corresponding authors were extracted from the WoS ‘Reprint Addresses’ field. For each of these three positions, we recorded the author's disciplinary profile using the classification described in §2.2.

The institutional sector was inferred from organization names: affiliations containing ‘university’, ‘school’, ‘college’ or similar terms were classified as academic; those containing ‘company’, ‘ltd’, ‘Inc’, ‘co.’, ‘corporation’, ‘GmbH’, etc., were classified as industry. At the author level, individuals listing only academic affiliations were labelled academic-only, those with only industry addresses were labelled industry-only and those appearing in both contexts were labelled academic–industry. At the paper level, a publication was classified as academic-only if all authors were academic; industry-only if all were from industry and academic–industry transdisciplinary if it included at least one academic-affiliated author and at least one industry-affiliated author. Papers in which no sector could be determined were labelled other/unknown.

The collaboration scope was quantified by extracting and normalizing country names from the tail of each affiliation string and counting the number of distinct countries and institutions per paper. Each paper was then classified as: local (all authors affiliated with a single institution), national (multiple institutions within one country) or international (affiliations spanning at least two countries). Countries were further grouped into world regions (Africa, Asia, Europe, North America, Oceania, South America) for regional summaries. To assess whether team-type composition varied with collaboration scale, we constructed contingency tables crossing collaboration scope (local, national, international) with team type (Tech-only, Bio-only, Bio–Tech) and applied chi-square tests of independence to the resulting tables. Similar cross-tabulations were built for world regions to check regional robustness. For all chi-square tests of independence, we reported Cramér's *V* as an effect-size measure of the association between categorical variables. Cramér's *V* was calculated from the chi-square statistic and sample size, adjusted for the dimensions of the contingency table and ranged from 0 (no association) to 1 (perfect association) with larger values indicating stronger associations. Note that affiliations captured the country of the employing organization, not authors’ personal nationalities.

### 2.5. Rule-based topic detection from textual metadata

To link team composition to research topics, we assigned papers to 13 biomimetic research domains. These domains were defined through a synthesis of topic structures reported in prior reviews of biomimetic research [15,20–22] and subsequently refined using an iterative corpus-based procedure. Specifically, we examined titles, abstracts, author keywords, Keywords Plus and Web of Science subject metadata to identify recurrent terminology patterns, resolve overlaps between candidate domains and calibrate topic boundaries, so that the final domain set captured the major research clusters represented in the dataset: *Robotics & Actuation*, *Sensors & Sensing*, *Surfaces & Wettability*, *Composites & Mechanical Metamaterials*, *Filtration & Separation*, *Medical & Tissue Engineering*, *Adhesion & Attachment*, *Optics & Photonics & Structural Color*, *Fluids & Drag Reduction*, *Energy Harvesting & Thermal Management*, *Self-healing & Damage Tolerance*, *Bioinspired Algorithms & Computation* and *Biofabrication & Manufacturing*. Each topic was defined by a curated set of core keywords, context keywords and exclusion patterns developed through iterative inspections and tests (supplementary material, S1).

The topic assignment used a weighted multi-source scoring system. For each paper, keyword matches in the title received a weight of 3.0, matches in the author keywords 2.5, matches in the WoS Keywords Plus field 1.5 and matches in the abstract 1.0. A paper was assigned to a topic only if its cumulative weighted score exceeded a topic-specific threshold. Papers could be tagged with more than one topic if they exceeded the threshold in multiple categories.

For the statistical analysis of team composition by topic, we collapsed team types into three groups (Tech-only, Bio-only, Bio–Tech) and cross-tabulated them with the 13 topics in a 13 × 3 contingency table. A chi-square test was used to assess whether team-type proportions differed systematically across topics.

### 2.6. Citation metrics

From WoS, we extracted the ‘Times Cited (WoS Core)’ count for each paper (performed on 13 November 2025) and computed a simple age-normalized citation rate,

(2.1) $$Citations\;Per\;Year\;(WoS\;CPY)\;=\frac{Times\;Cited\;(WoS\;Core)\;}{2025-publication\;year+1}.$$

To test whether team composition was associated with citation metrics after controlling for publication age, we fitted a negative binomial regression at the paper level with raw WoS citation count as the response variable and team type and publication year as predictors (2004–2020; Bio–Tech as reference category). IRRs quantified the relative citation rate of each team type. Papers published after 2020 were excluded because their short citation windows did not yet allow reliable comparison.

To additionally correct for both age and field effects, we complemented this measure with the US National Institutes of Health (NIH) Relative Citation Ratio (RCR). The RCR was an article-level metric that quantified citation impact while normalizing for both field and time [23]. It compared a paper's observed citation rate with an expected citation rate derived from the citation behaviour of papers in its co-citation-defined field and publication year, using NIH-funded publications as the benchmark. In this framework, an RCR of 1.0 indicated citation performance equal to the benchmark expectation, whereas values above or below 1.0 indicated citation performance higher or lower than that field- and year-normalized expectation (e.g. an RCR of 2.0 reflected approximately twice the expected citation rate). Papers were matched to NIH iCite records via PMIDs and DOIs, and the RCR together with its provisional flag and field citation rate were imported. For analyses of age- and field-normalized impact, we used all papers with a non-missing RCR and compared those RCR values across team types using a Kruskal–Wallis test, followed by Dunn's post hoc pairwise comparisons with Bonferroni correction. As a complementary parametric check, we fitted an ordinary least squares (OLS) regression (RCR ∼ TeamType + Year) to estimate effect sizes while controlling for publication year.

All above-mentioned statistical calculations were performed in Python 3.12.0 using the statsmodels package (v. 0.14.5; [24]); non-parametric tests and post hoc comparisons used SciPy (v. 1.13.1; [25]) and scikit-posthocs (v. 0.12.0; [26]).

### 2.7. Linking to biological models

We linked the author disciplinary data from this study to an existing database of biological models compiled in the previous work on taxonomic patterns in biomimetics [14]. In the previous study, GPT-4o-2024-08-06 was used as a structured information-extraction tool applied to the titles, keywords and abstracts of 74 359 WoS records. The workflow involved two sequential prompts: first, a model-extraction prompt identified organisms or organism groups explicitly used as sources of biological inspiration, while excluding incidental examples or experimental organisms not used as biomimetic models; second, a taxonomy-assignment prompt classified each extracted model from kingdom to species level and returned ‘NA’ where taxonomic resolution was ambiguous. Records were processed individually to reduce cross-record contamination and improve output consistency. To guard against erroneous or fabricated outputs, both steps were manually validated against a reference standard before use, each exceeding a predefined 95% accuracy threshold (model extraction: 95.9%; taxonomy assignment: 95.1%, verified against taxonomic sources including GBIF [27], OneZoom Tree of Life Explorer [28] and Bioinspire-Explore [29]), followed by a consistency-cleaning pass over all extracted models that combined automated (Python-based) flagging with manual correction. Full details, prompts and validation records are reported in [14]. Nevertheless, because the source mining relied on titles, keywords and abstracts rather than full texts, and because some biological model names are broad or ambiguous, residual uncertainty remained and was considered a limitation of the present analysis.

For the present analysis, we matched this model database to our author-affiliation corpus primarily via DOI; where DOIs were missing, we used title-based matching. This cross-linking yielded the subset of papers for which both team composition and at least one recognized biological model were available, and all analyses involving biological models were restricted to this intersection.

From the model database, we imported for each intersection paper the list of biological models at their finest available taxonomic resolution. We then derived four summary metrics for each team category (Tech-only, Bio-only, Bio–Tech, Other/Unknown):

– the proportion of papers that contained at least one identifiable biological model,
– the number of unique taxa per 100 model-bearing papers, where ‘unique’ denoted distinct taxa at their finest reported taxonomic rank,
– the distribution of taxonomic resolution, measured as the share of models specified at species, genus, family, order, class, phylum or kingdom level, and
– the distribution of model recurrence, classified by global frequency as: core (more than 20 occurrences across the entire reference corpus), frequent (6–20 occurrences), occasional (2–5 occurrences) or rare (singletons).

To examine whether biological model use was concentrated within specific research domains, we cross-tabulated the biological models with the 13 topic domains defined in §2.5. Because a single paper might cite more than one biological model and might also be assigned to more than one topic, analyses were performed on paper–model–topic links; the topic-tagged model corpus comprised 12 439 such links. For each topic–model combination, enrichment lift was calculated as the share of that model among all model links within a given topic divided by its share across the full topic-tagged model corpus. Values greater than 1 therefore indicated models that were over-represented within a topic relative to their corpus-wide use. We used one-sided Fisher's exact tests to assess enrichment and applied Benjamini–Hochberg correction for multiple testing.

### 2.8. Validation of automated classification

To assess the accuracy of the automated discipline-classification pipeline, 30 papers (with a total of 178 author–paper affiliation records) were sampled with quotas stratified by team type and manually validated by the first author. Agreement between automated and manual discipline labels reached 99.4% (177/178 records); agreement on paper-level team type was 100% (30/30 papers). Full validation details and results are reported in supplementary material, S2. We therefore considered the automated classification sufficiently reliable to serve as the basis for this study.

## 3. Results and discussion

### 3.1. Field-wide team composition

Of the 74 407 publications in the corpus, 38.3% (28 514 papers) were written by Bio–Tech teams; Tech-only teams account for 49.2% (36 609 papers) and Bio-only teams for 8.2% (6132 papers) (figure 1a). Full results of the affiliation matching records are provided in supplementary material, S3.

**Figure 1.**
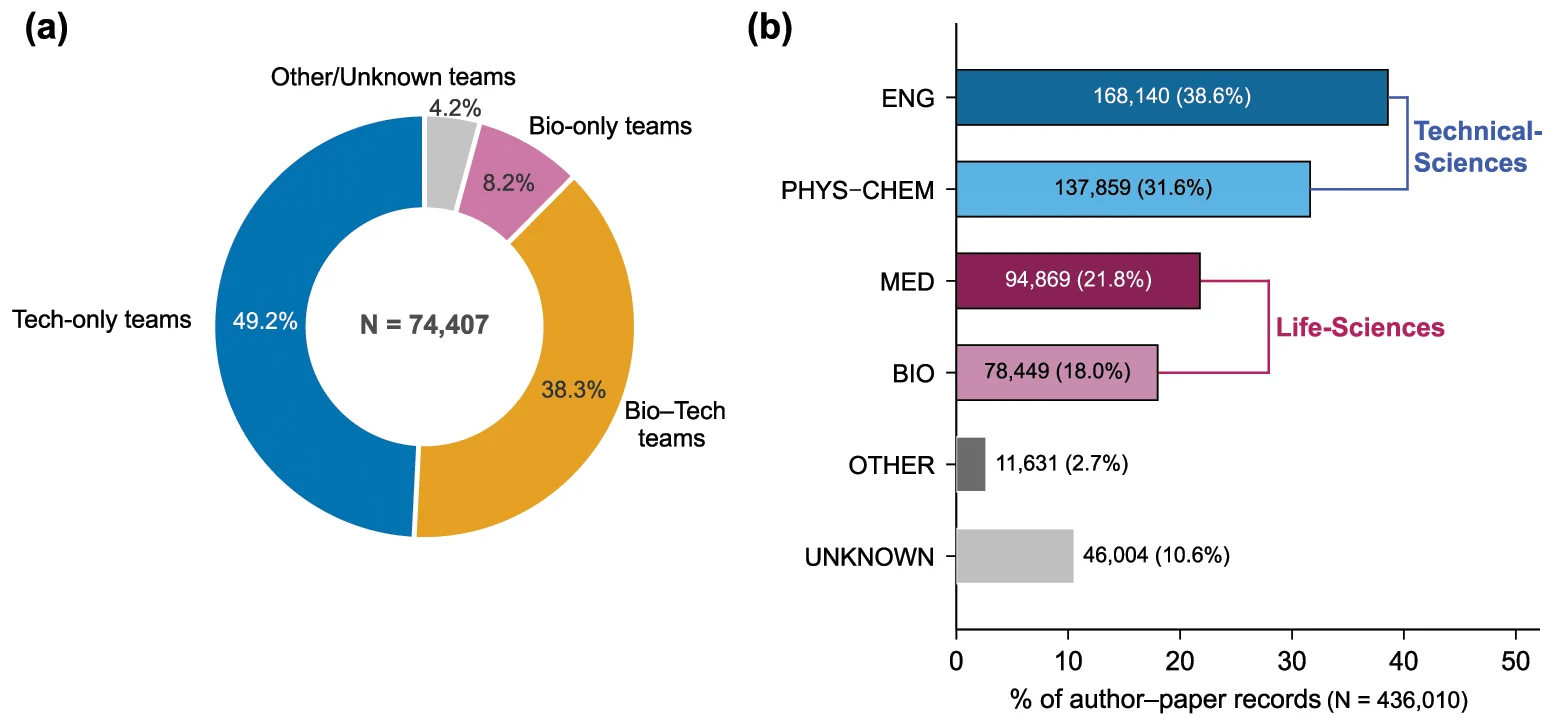
Field-wide distribution of team types. (a) Paper-level team composition. The ‘Other/Unknown teams’ here includes both papers with only OTHER/UNKNOWN author labels and papers assigned through the uncertain subject-category fallback. (b) Author-level disciplinary backgrounds. Because authors may hold affiliations spanning multiple categories, counts are not mutually exclusive.

The author-level distribution shows amplified asymmetry between technical and biological expertise (figure 1b). Among the total 436 010 author–paper affiliation records, 390 006 (89.4%) could be assigned to at least one disciplinary background; of the assigned records, Technical-Sciences authors clearly dominate with 305 999 records (78.4%). The pattern is clear: technical backgrounds are ubiquitous, while biological and medical expertise forms a substantial contributor pool, with medical backgrounds exceeding purely biological backgrounds.

Within Bio–Tech teams, hybrid authors (those with both Life-Sciences and Technical-Sciences backgrounds) were the single largest category in all key authorship positions: 46.6% of first authors, 49.9% of last authors and 53.5% of corresponding authors. Authors with exclusively technical affiliations were the second most common group (32.0%, 29.5% and 26.4%, respectively), while those with exclusively biological affiliations remained stable at approximately 18% across all three positions. Individuals who combine biological and technical affiliations are thus disproportionately represented in leadership roles. One interpretation is that such hybrid profiles reflect a bridging function, in which these authors coordinate across disciplinary boundaries. However, the pattern may also reflect the prominence of inherently hybrid fields, such as biomedical engineering, whose institutional labels span both domains.

### 3.2. Growth trajectories and the rise of Bio–Tech collaboration

Biomimetics publications have shown sustained growth over the past two decades, from 651 papers in 2004 to 9159 papers in 2025. Decomposing this growth by team type reveals divergent trajectories (likelihood-ratio test, *χ*² = 79.5, *df* = 2, *p* < 0.0001) (figure 2a,b). In absolute terms, Tech-only teams dominate throughout, rising from 381 papers in 2004 to 4323 in 2025, corresponding to an annual growth rate of approximately 10.6% (IRR = 1.106, 95% CI 1.101–1.110). Bio–Tech teams follow a steeper curve, increasing from 162 to 3624, a 22-fold expansion at approximately 15.0% yr^−1^ (IRR = 1.150, 95% CI 1.145–1.156). Bio-only teams grew at approximately 14.7% yr^−1^ (IRR = 1.147, 95% CI 1.139–1.154), but their absolute volume remains much smaller in comparison (figure 2a). These divergent growth rates produce a pronounced compositional shift: in the mid-2000s, Tech-only teams accounted for roughly 64% of categorized papers and Bio–Tech teams for about 27%; by the early 2020s, these shares had converged to approximately 48% and 42%, respectively (figure 2b). This pattern signals a transition from a predominantly engineering-driven enterprise to one in which interdisciplinary collaboration has become normative for a large and growing fraction of publications.

**Figure 2.**
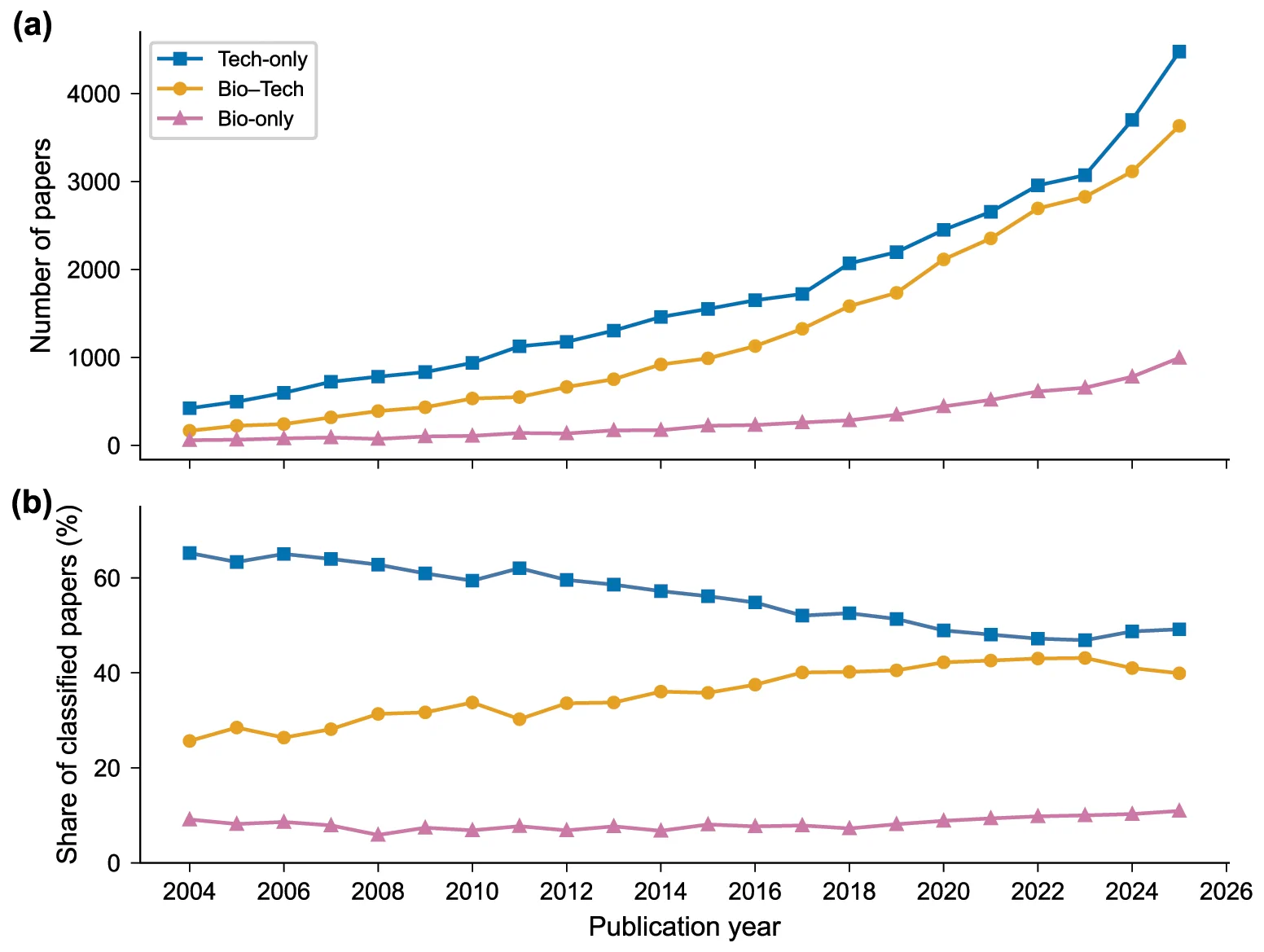
Temporal dynamics of team composition (2004–2025). (a) Absolute yearly paper counts of Bio–Tech teams, Tech-only teams and Bio-only teams. (b) Yearly shares (%) of publication volume across Bio–Tech teams, Tech-only teams and Bio-only teams.

### 3.3. Sectoral and geographic architecture

In our dataset, the organizational sector reveals a clear imbalance. Of the 74 407 corpus papers, 89.6% list only academic institutions, academic–industry collaborations account for 3.6% and purely industrial papers number just 0.1% (figure 3a). The imbalance is even starker at the author level: of 436 010 author-paper records, only 1.1% carry any industry affiliation, and fewer than half of those authors (1833) hold joint academic–industry backgrounds. The near-absence of industry from published biomimetics is consistent with patterns observed across disciplines: Abramo *et al.* [30] found similarly low rates (approximately 3%) of university–industry co-publication in their overall Italian research publication dataset; Perkmann *et al.* [31] documented in a systematic review that much academic–industry engagement occurs through consulting, contract research and other collaboration channels that often do not result in co-authored publications. This under-representation also reflects a methodological boundary of our approach: whereas the academic/industry sector can be inferred from organization names, the disciplinary classification depends on the departmental or sub-unit structure of an affiliation, which company names rarely provide, so industry-affiliated authors are disproportionately assigned to the Other/Unknown category (figure 3a). A fuller account of industrial engagement would require complementary sources such as patents and technology disclosures. Existing patent-based studies indicate that bio-inspired innovation is active and growing in the patent record [32–34], which our publication-based analysis could not capture. We therefore interpret the sectoral results as a map of industry visibility within the published literature, rather than as a complete measure of industry participation in biomimetic and bioinspired innovation.

**Figure 3.**
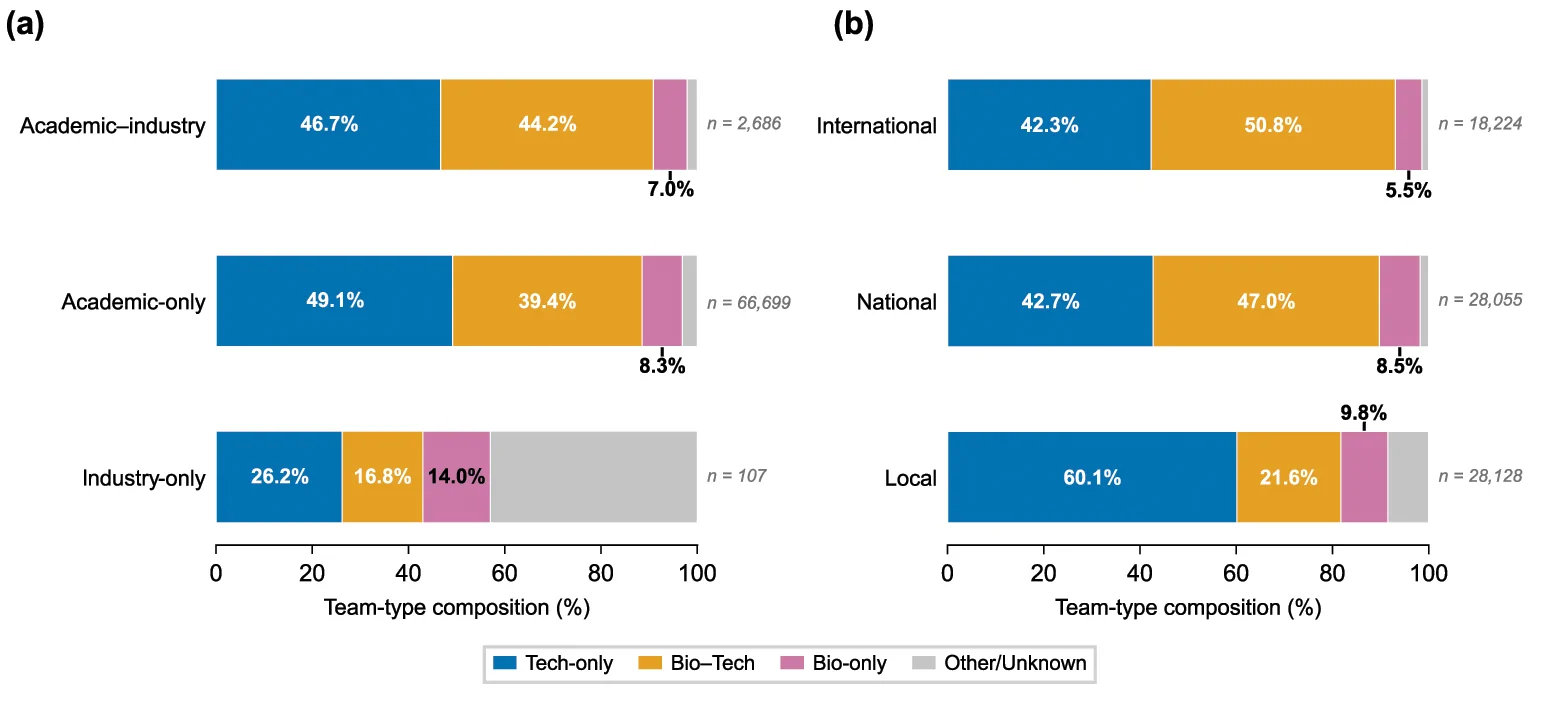
Team-type composition by (a) institutional sector and (b) collaboration scope. Bars show the percentage of papers in each category by team type; sample sizes are given to the right of each bar.

By geographic scope, 24.5% of papers involve authors from multiple countries (international), 37.7% involve multiple institutions within a single country (national) and 37.8% originate from a single institute (local). Team composition shifts with collaboration scope (*χ*² = 4670.6, *df* = 4, *p* < 0.0001, Cramér's *V* = 0.18): Tech-only teams are over-represented in local (single-institution) settings, where they account for 60.1% of records; whereas Bio–Tech teams are over-represented in broader collaboration networks, surpassing Tech-only teams at both the national and international scale (figure 3b). Across world regions, Asia (44 888 papers) shows 50.4% Tech-only and 39.2% Bio–Tech; Europe (22 696) shows 47.1% and 41.1%; North America (17 407) is the most interdisciplinary, with Bio–Tech (45.0%) exceeding Tech-only (43.3%).

Focusing on the three largest producers of biomimetic research papers—China (30 083 papers), the USA (15 648) and Germany (4779)—reveals distinct national pathways to interdisciplinary biomimetics (figure 4a–f). Across our corpus, China remains overall technology-dominant, with Tech-only teams accounting for 50.8% of papers and Bio–Tech teams for 39.6%, alongside a predominantly domestic collaboration structure (78.6% within-country). By contrast, the USA shows near-parity between Bio–Tech (45.8%) and Tech-only teams (42.3%), with a substantially higher share of international co-authorship (41.1%). Germany also approaches parity in team composition, with Bio–Tech teams representing 41.2% of papers, but stands out for its strongly international collaboration profile, with 57.3% of papers involving cross-border co-authorship.

**Figure 4.**
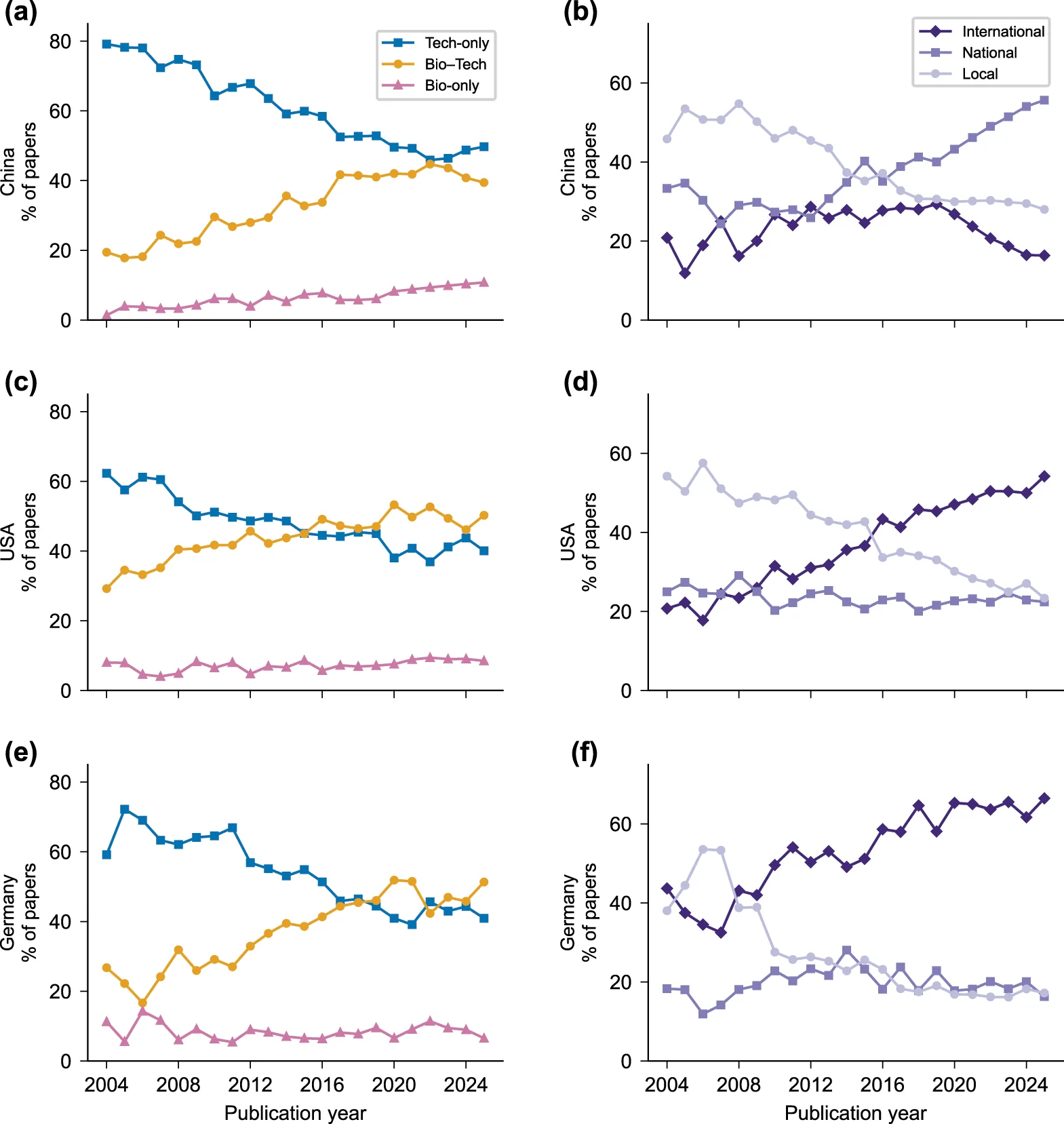
National profiles of the three leading countries by biomimetics publication output. For each country: team-type shares over time (left) and collaboration scope shares over time (right). (a,b) China; (c,d) USA; (e,f) Germany.

Year-by-year trends reveal further divergence in how these national profiles have evolved. In all three countries, the annual share of Bio–Tech teams has increased over time (figure 4a,c,e). However, the USA and Germany show a pattern of interdisciplinary teams surpassing Tech-only teams in recent years, whereas China remains consistently led by Tech-only teams on an annual basis. The collaboration trends are similarly differentiated: international collaboration has expanded markedly in both the USA and Germany and exceeded domestic collaboration in each; whereas China has followed a different trajectory, with international collaboration declining in recent years (figure 4b,d,f). Notably, Van Noorden [35] reported that China ranks among the most interdisciplinary countries globally when measured by cross-disciplinary citation patterns. Yet, in our affiliation-based study of biomimetic publications, China's interdisciplinary capacity appears to have been built primarily through domestic multi-institutional networks. This pattern is consistent with earlier evidence of lower international collaboration rates among Asian countries [36], though it contrasts with more recent findings that Chinese science overall has expanded both domestic and international collaborations at rates exceeding most established nations [37].

### 3.4. Citation patterns across team types

The preceding sections establish that biomimetics is asymmetric in team composition. We now assess whether this asymmetry has consequences for citation impact. Across the corpus (2004–2020), Bio–Tech teams achieve the highest mean WoS citations per year (WoS CPY) (5.96), followed by Tech-only (5.59) and Bio-only (4.45) (figure 5a). A negative binomial regression with publication year as covariate confirmed that these differences are significant: compared with Bio–Tech teams, Tech-only papers receive 15.5% fewer citations (IRR = 0.845, 95% CI 0.824–0.867, *p* < 0.0001) and Bio-only papers 31.2% fewer (IRR = 0.688, 95% CI 0.652–0.726, *p* < 0.0001). Total citation volume (figure 5b) is primarily driven by publication volume, as Tech-only teams produce more papers overall; however, Bio–Tech teams approach or match Tech-only teams in total citations despite producing fewer papers, consistent with their higher per-paper citation intensity.

**Figure 5.**
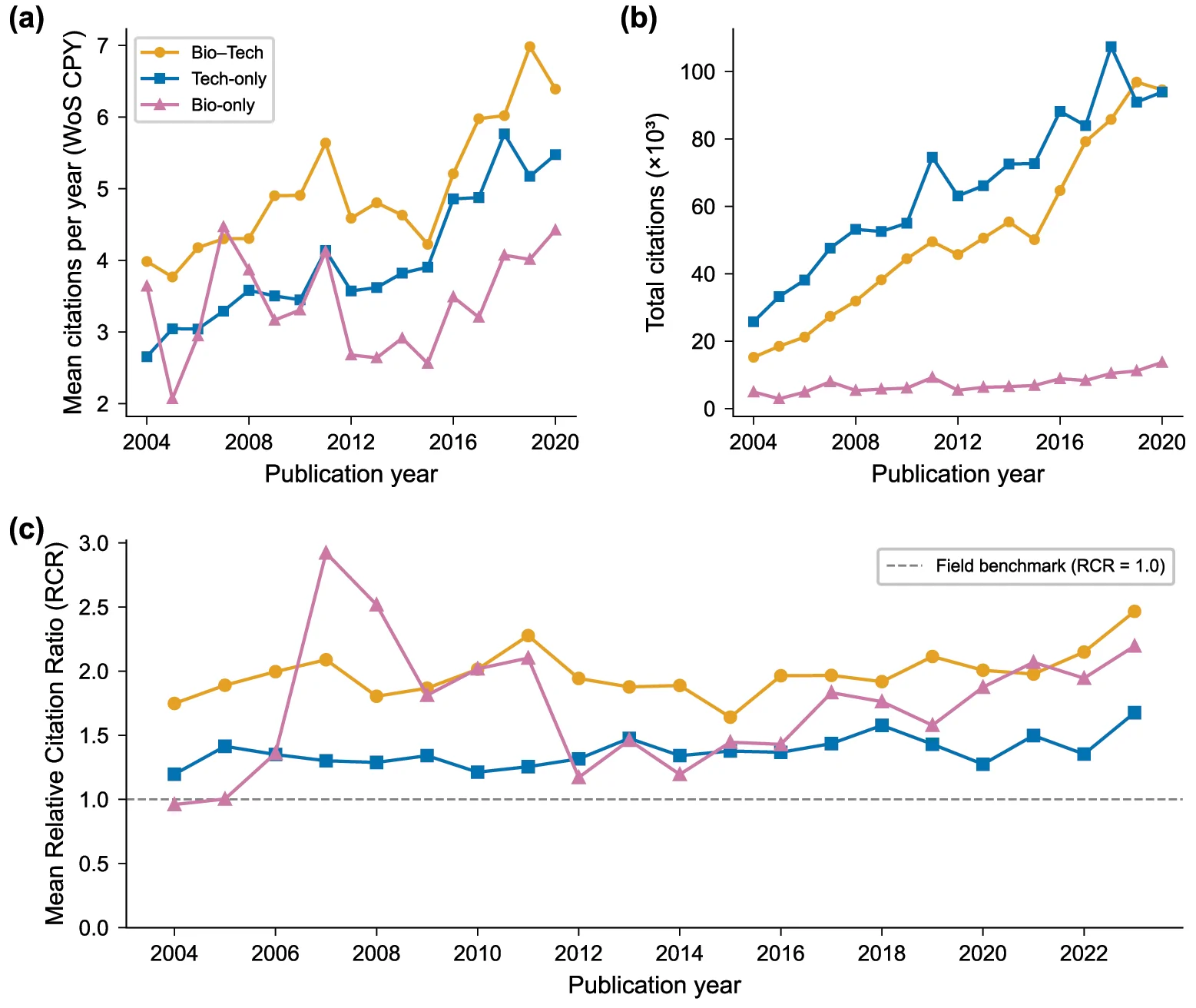
Citation performance by team type. (a) Mean WoS CPY and (b) total citation volume (×10³) received by each team type between 2004 and 2020; publications after 2020 are excluded because their short citation windows do not yet allow reliable comparison. (c) Mean RCR by team type for papers with mature citation windows (2004–2023; *n* = 23 819). The dashed line marks the NIH field benchmark (RCR = 1.0), at which a paper accrues citations at exactly the rate expected for its field and year.

For field-normalized impact, we analysed NIH RCR values for 23 819 papers with mature citation windows (2004–2023). Coverage varies by team type (Bio–Tech 44.2%, Bio-only 43.0%, Tech-only 24.6%), reflecting PubMed's biomedical focus. Bio–Tech teams achieve a mean RCR of 2.07 (median 1.27), roughly twice the field expectation; Bio-only teams also perform above baseline (mean 1.87, median 1.15); Tech-only teams average 1.44 (median 0.82), with over half falling below the field benchmark (figure 5c). RCR values differ significantly among team types (Kruskal–Wallis *H* = 801.7, *p* < 0.0001), with all pairwise differences significant after Bonferroni correction (Dunn's test: Bio–Tech vs Tech-only, *p* < 0.0001; Bio–Tech vs Bio-only, *p* = 0.005; Tech-only vs Bio-only, *p* < 0.0001). An OLS regression controlling for publication year yielded consistent results: Tech-only papers score 0.60 RCR points lower than Bio–Tech (*β* = −0.596, 95% CI −0.668 to −0.525, *p* < 0.0001), and Bio-only papers 0.21 points lower (*β* = −0.208, 95% CI −0.329 to −0.087, *p* < 0.0001). The Bio–Tech citation advantage is also temporally stable, with mean RCR consistently exceeding that of Tech-only across all years from 2004 to 2023.

We cannot rule out that the Bio–Tech citation advantage partly reflects topic selection rather than a pure compositional effect of interdisciplinarity. Nevertheless, the convergence of raw and field-normalized metrics, confirmed by regression models controlling for publication year, provides evidence that integrating biological and technical expertise is associated with higher citation performance in biomimetics.

### 3.5. Topic-specific disciplinary profiles

Having established that Bio–Tech teams often achieve higher citation performance, we now examine how team composition varies across research domains. Of the 74 407 corpus papers, 24 488 (32.9%) were assigned to at least one of the 13 application-domain topics; the remainder fell below all topic-specific score thresholds. The topic-assigned subset mirrors the full corpus in team-type distribution (Tech-only 46.4%, Bio–Tech 43.3%, Bio-only 7.8%), confirming that it is compositionally representative.

Topic volume differs across domains, spanning nearly two orders of magnitude from *Medical & Tissue Engineering* (8461 papers) to *Bioinspired Algorithms & Computation* (182 papers) (figure 6). Team composition is topic-dependent with distinct disciplinary profiles across domains. On one end, five engineering-dominated topics—*Bioinspired Algorithms & Computation*, *Energy Harvesting & Thermal Management*, *Robotics & Actuation*, *Sensors & Sensing* and *Composites & Mechanical Metamaterials*—exceed 70% Tech-only, with Bio-only teams contributing fewer than 3% of papers in each case. On the other end, *Medical & Tissue Engineering* inverts the pattern: Bio–Tech teams account for 66% and Bio-only for 16%, leaving Tech-only teams at 17%. Between these poles, several topics occupy an intermediate position: *Fluids & Drag Reduction*, *Optics & Photonics & Structural Color*, *Surfaces & Wettability*, *Self-healing & Damage Tolerance*, *Filtration & Separation* still show Tech-only majorities of 60%–68%, but also include a substantial Bio–Tech component of approximately 23%–32% of papers. *Adhesion & Attachment* shows the closest-to-balanced distribution, with Tech-only and Bio–Tech teams contributing almost equal shares. We therefore interpret these as domains in which interdisciplinary collaboration is clearly present, although it has not become the majority team structure. This gradient tracks the degree to which a topic's core questions require direct engagement with biological expertise. A chi-square test confirms that team composition is significantly associated with research topic (*χ*² = 5964.4, *df* = 24, *p* < 0.0001), with a moderate effect size (Cramér's *V* = 0.32).

**Figure 6.**
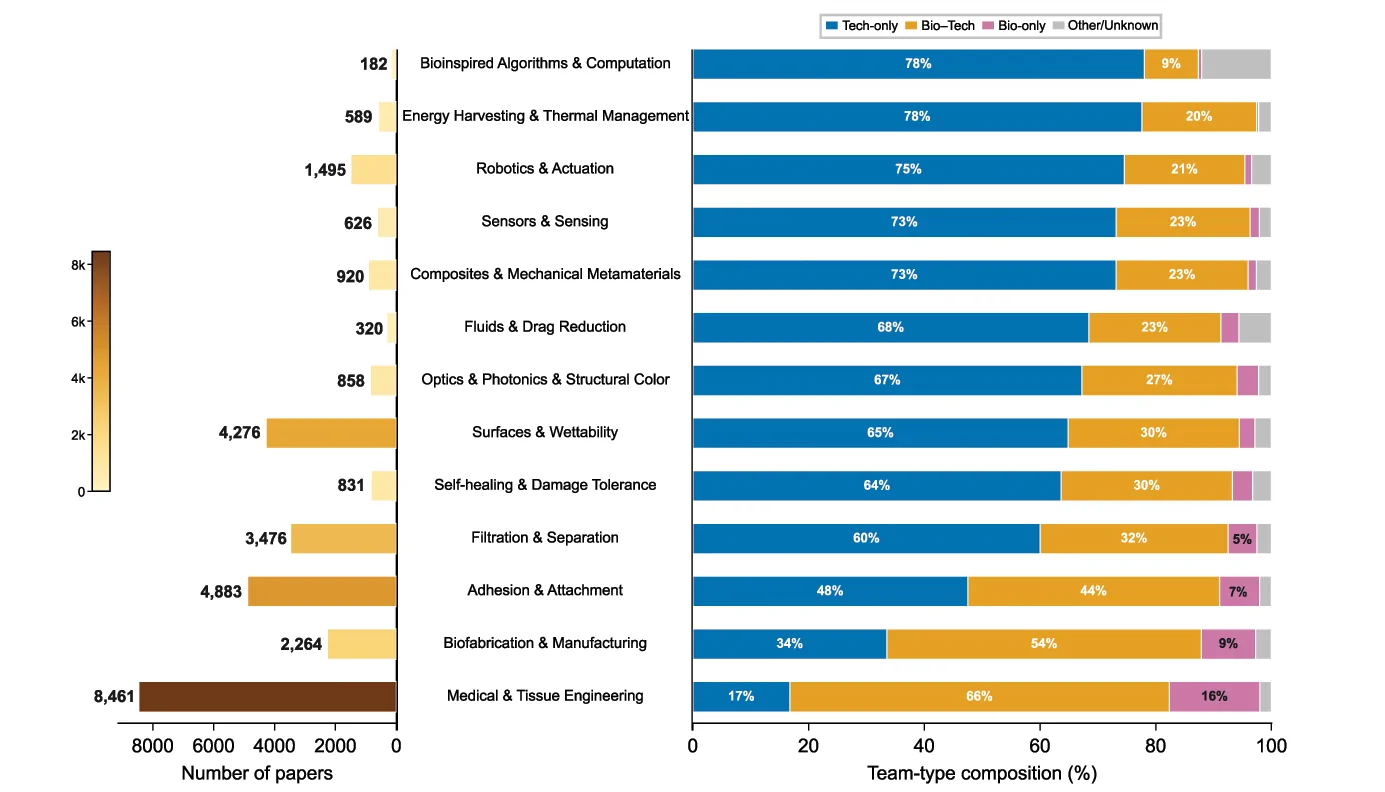
Topic-specific disciplinary profiles of biomimetics. Left: number of papers per application domain; colour encodes volume. Right: team-type composition within each topic. Topics are ordered by descending Tech-only teams’ share (%). Only shares greater than or equal to 5% are labelled in the figure (excluding the ‘Other/Unknown’ category).

### 3.6. Biological model use and the role of biological expertise

Linking team composition to the biological models declared in each paper reveals how compositions of expertise translate into different organism choices. Across the full corpus, approximately one-third of papers (24 525 papers; 33.0%) explicitly reference at least one biological model. Bio–Tech teams show the highest propensity to mention a model (35.4% of all Bio–Tech papers), followed by Tech-only (32.9%) and Bio-only teams (30.4%). Although these proportions differ significantly (*χ*² = 74.9, *df* = 2, *p* < 0.0001), the effect size is negligible (Cramér's *V* = 0.03), indicating that the likelihood of referencing a biological model is largely independent of team type. In absolute numbers, Tech-only teams produce the largest share of model-bearing papers (11 318 papers; 46.2%), followed by Bio–Tech (9758; 39.8%) and Bio-only (1777; 7.2%). Most papers focus on a single organism, and fewer than 1% mention four or more organisms.

The three team types differ markedly in how broadly they sample from the available repertoire of biological models (figure 7). Bio-only teams draw on 24.5 distinct models per 100 model-bearing papers, roughly twice the rate of Bio–Tech (13.2) and Tech-only (11.8) teams. This diversity gap is echoed in rare-model usage: 8.1% of organism references in Bio-only papers are singletons (taxa appearing in only one paper in the entire corpus), compared with 5.4% for Bio–Tech and 4.4% for Tech-only. Bio-only teams thus appear more willing to introduce novel organisms into the biomimetic repertoire.

**Figure 7.**
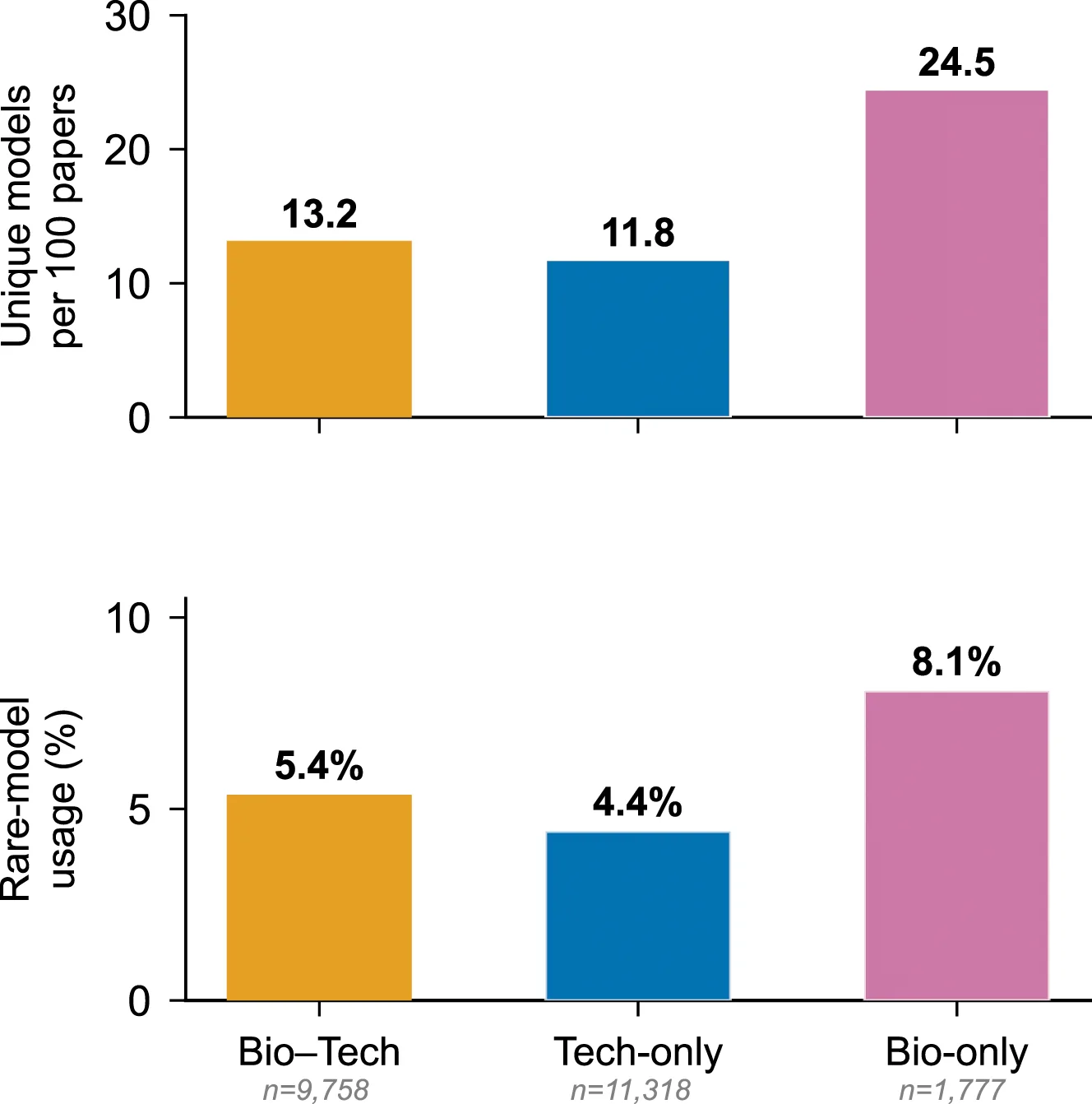
Biological model diversity by team type. Unique biological models per 100 model-bearing papers (top) and percentage of model references that are singletons (bottom).

When Bio-only teams are decomposed into their underlying Life-Sciences components, those composed exclusively of BIO authors (*n* = 562 papers) achieve species-level identification at 38.1%, nearly twice the rate of those with purely MED authors (*n* = 937). The same divergence extends to model novelty: Bio-only teams with solely BIO authors draw on organisms with a mean global frequency of 80 and produce singletons at 30.4%, compared with 134 and 15.4% for teams with solely MED authors. This pattern is not confined to Bio-only teams: within Bio–Tech collaborations, teams whose biological component consists exclusively of BIO authors (*n* = 4522) reach 26.8% species-level identification, whereas those with exclusively MED authors (*n* = 2454) achieve 19.8%. Across all fine-grained team configurations, species-level precision tracks the presence of BIO authors, not Life-Sciences authors in general.

We next examined whether biological-model use is itself topic-specific, testing which model labels are over-represented within each domain relative to the topic-tagged model corpus. We interpreted over-representation only for model labels with lift > 1 and false discovery rate (FDR)-adjusted *q* < 0.001. Model use was topic-specific and broadly followed the disciplinary gradient shown in figure 6. In the technology-dominated topics, over-represented models reflected the engineering problem structure: *Bioinspired Algorithms & Computation* was enriched for swarm-intelligence metaphors, including ant (8/110 model links; 7.3% of the topic's model occurrences; lift = 82.2) and ant colony (5/110; 4.5%; lift = 113.1); *Robotics & Actuation* was enriched for locomotion and actuation models such as inchworm (30/869; 3.5%; lift = 11.9), snake (26/869; 3.0%; lift = 9.8), octopus (32/869; 3.7%; lift = 4.2) and fish (34/869; 3.9%; lift = 4.3); whereas *Composites & Mechanical Metamaterials* was dominated by nacre (239/572; 41.8%; lift = 14.5).

Intermediate surface- and structure-oriented domains converged on canonical functional models: *Adhesion & Attachment* was dominated by mussel (640/2495; 25.7%; lift = 3.4) and gecko (244/2495; 9.8%; lift = 3.9), *Surfaces & Wettability* by lotus leaf (269/2153; 12.5%; lift = 3.8), *Fluids & Drag Reduction* by shark (86/226; 38.1%; lift = 32.0) and *Optics & Photonics & Structural Color* by moth (45/505; 8.9%; lift = 16.8), morpho butterfly (21/505; 4.2%; lift = 16.2) and beetle (41/505; 8.1%; lift = 11.5).

By contrast, the Bio–Tech-dominated *Medical & Tissue Engineering* domain was enriched for biomedical tissue, cell and extracellular-matrix models, including bone (268/2554; 10.5%; lift = 3.0), collagen (93/2554; 3.6%; lift = 2.5), red blood cell (78/2554; 3.1%; lift = 3.9) and cartilage (76/2554; 3.0%; lift = 3.5). *Biofabrication & Manufacturing* showed a related but broader pattern, combining tissue/material models with fabrication-oriented biological systems such as diatoms (12/837; 1.4%; lift = 4.9) and magnetotactic bacteria (7/837; 0.8%; lift = 8.0).

Topic-specific disciplinary profiles are thus accompanied by corresponding concentrations of biological-model use: physically and engineering-oriented domains repeatedly invoke small sets of iconic, functionally matched exemplars, whereas medically oriented work centres on tissue- and cell-associated models. This pattern is consistent with field-level evidence that biomimetic research relies heavily on a relatively conservative set of biological models [14], although the association is descriptive and should not be interpreted causally.

## 4. Conclusions and outlook

This study set out to answer the question: who is active in biomimetic research? By classifying 436 010 author–paper affiliation records across 74 407 publications spanning two decades, we provide the first field-scale map of the disciplinary composition, collaborative structure, geographic distribution and their associations with citation performance and biological model use in biomimetic research. Three principal findings emerge.

First, biomimetics has undergone a structural transition. What was once dominated by engineering sciences has now become a field in which interdisciplinary teams are a major and growing force for publication output. This transition is geographically diverse: as the world's largest producer of biomimetics publications, China has built its interdisciplinary capacity primarily through domestic multi-institutional networks; whereas Germany's interdisciplinary output is achieved overwhelmingly through international partnerships, illustrating that national research systems shape distinct organizational pathways to interdisciplinary biomimetics. While biomimetics is getting more interdisciplinary in team composition, biological expertise remains numerically minor in the authorship pool. At the same time, the near-absence of industry representation from the published literature raises questions about whether biomimetics has progressed toward transdisciplinary engagement with industrial and societal actors.

Second, this disciplinary asymmetry has consequences. Integrating biological and technical expertise is consistently associated with better citation performance. Team composition also varies dramatically across research topics—revealing domains where biological input is already structurally embedded (e.g. tissue engineering or biofabrication) and domains where biological expertise is almost entirely absent (e.g. robotics, algorithms, or energy harvesting). These ‘cold spots of interdisciplinarity’, concentrated in engineering-driven application areas, represent the opportunity spaces where targeted inclusion of biological expertise could reshape practice.

Third, biological expertise is not interchangeable. Distinguishing organismal biologists from medically trained researchers reveals that taxonomic precision and organism novelty are specifically associated with organismal biology backgrounds. This pattern suggests that it is organismal-biology competence (e.g. ecological, zoological, botanical), rather than biological expertise in general, that introduces taxonomic breadth into biomimetic research.

These conclusions should be interpreted in light of several limitations. The corpus is delimited by terminology rather than by conceptual adherence: because the labels ‘biomimetics’, ‘biomimicry’, ‘bio-inspired’ and ‘bioinspiration’ are applied inconsistently in publication practice [5], we retained an inclusive corpus and did not judge whether each paper meets a strict definition of biomimetics. This boundary affects the team composition we report: a decomposition of the corpus by search term indicates that publications retrieved by ‘bio*inspir*’ terms are more strongly technology-dominated (i.e. have a higher proportion of Tech-only teams) than those retrieved by ‘biomim*’ terms. Discipline is inferred from affiliation labels at the time of publication rather than training history (e.g. a biologist housed in an engineering department will be recognized as an engineer in our study); in addition, inherently interdisciplinary units such as biomedical engineering, bioengineering and biological engineering are counted under both BIO and ENG, so part of the observed hybrid profile may reflect institutional labelling rather than collaboration between separately identifiable biological and technical specialists. Authorship captures only credited participation, not informal or early-stage contributions. The corpus is restricted to WoS-indexed papers and therefore under-represents industrial activity captured through patents or commercial products. Finally, the observed associations cannot establish causality: topic selection, institutional resources and field norms may jointly shape both team composition and outcomes.

Looking ahead, three future research directions would add the most value. First, combine bibliometrics with qualitative evidence (surveys and interviews) to resolve mechanisms—when biological experts enter projects, which decisions they influence and how credit is assigned. Second, link publications to patents, products or other non-academic pathways to quantify where and how industry engagement enters the innovation pipeline beyond the scholarly record. Third, use the topic-level disciplinary profiles established here as a baseline for asking whether, and under what conditions, funding instruments, training programmes or institutional reforms are associated with shifts in team composition, particularly in engineering-dominated domains.

Overall, this work provides a reference map of the field's ‘human architecture’. It therefore offers a baseline for evaluating future efforts to strengthen the biological foundations of biomimetic innovation. Our evidence suggests that interdisciplinarity in biomimetics should not be treated as a binary attribute to be maximized, but as a compositional variable to be designed: what matters is not simply whether biological expertise is present, but what kind is involved and into which research domains it is directed.

## Supplementary Material



## Data Availability

All data needed to evaluate the conclusions in the paper are present in the paper and/or the supplementary material. Supplementary material is available online [38].

## Declaration of AI use

For the present manuscript, ChatGPT (OpenAI) was used only for language editing and readability improvement, including grammar, spelling, word form, tense and sentence-flow checks. All suggested edits were reviewed and revised by the first author (J.Z.). ChatGPT was not used to design the study, classify author affiliations, generate data, perform statistical analyses, produce figures or develop any scientific interpretation. Representative examples of the language edits are provided in supplementary material, S4. Separately, this study re-uses a biological-model database generated in our previous work [14], in which GPT-4o-2024-08-06 was used as a structured information-extraction tool to identify biological models from titles, keywords and abstracts, and to support taxonomic assignment. That workflow included manual validation, consistency checks and cleaning before analysis. We have described this reuse explicitly in §2.
