# Supplementary material for: Who mimics nature? Disciplinary asymmetry in biomimetic and bioinspired research teams
Source: J R Soc Interface. 2026 Oct 7;23(243):20260324. doi: 10.1098/rsif.2026.0324 (PMC13641953; doi:10.1098/rsif.2026.0324)
Supplement: Supplementary file 1 [file rsif.2026.0324.zip › rsif.2026.0324/rsif20260324_si_004.pdf]

ChatGPT-5.5 (OpenAI) was used only for language editing and readability improvement in the present manuscript. The examples below illustrate the type of corrections made. These edits concern grammar, spelling, word form, tense and sentence flow only, and did not introduce data, analyses, interpretation or scientific content.

**Raw author input**	**ChatGPT suggested output (and accepted by the author)**	**Section in the manuscript**
"The classification followed a multi-layer hierarchical rule-based pipeline implemented via Python with the aim to ensure that (i) explicit, auditable rules control known edge cases, while (ii) generalisable patterns cover the majority of affiliation strings."	"The classification followed a multi-layer hierarchical rule-based pipeline implemented in Python with the aim of ensuring that (i) explicit, auditable rules control known edge cases, while (ii) generalisable patterns cover the majority of affiliation strings."	2.2
"However, the pattern may also reflect the prominent of inherently hybrid fields, such as biomedical engineering, whose institutional labels span both domains."	"However, the pattern may also reflect the prominence of inherently hybrid fields, such as biomedical engineering, whose institutional labels span both domains."	3.1
"In the Bio–Tech teams, hybrid authors (those with both Life-Sciences and Technical-Sciences backgrounds) are the single largest category in all key authorship positions: 46.6% of first authors, 49.9% of last authors, and 53.5% of corresponding authors."	"Within Bio–Tech teams, hybrid authors (those with both Life-Sciences and Technical-Sciences backgrounds) were the single largest category in all key authorship positions: 46.6% of first authors, 49.9% of last authors, and 53.5% of corresponding authors."	3.1
"Discipline is inferred from affiliation labells at the time of publication rather than training history (e.g., a biologist housed in an engineering department will be recognised as an engineer in our study); and authorship captured only credited participation, not informal or early-stage contributions."	"Discipline is inferred from affiliation labels at the time of publication rather than training history (e.g., a biologist housed in an engineering department will be recognised as an engineer in our study); and authorship captures only credited participation, not informal or early-stage contributions."	4
